# Repeatability of Rapid Human Cardiac Phosphorus MRSI (^31^P-MRSI) Using Concentric Ring Trajectory Readouts at 7T

**DOI:** 10.1002/mrm.70220

**Published:** 2025-12-08

**Authors:** Ferenc E. Mózes, William T. Clarke, Andrew Tyler, Jabrane Karkouri, Fabian Niess, Jack J. J. J. Miller, Christopher T. Rodgers, Wolfgang Bogner, Ladislav Valkovič

**Affiliations:** 1 Oxford Centre for Clinical Magnetic Resonance Research, Radcliffe Department of Medicine, Division of Cardiovascular Medicine, University of Oxford, Oxford, UK; 2 Oxford Centre for Integrative Neuroimaging, Nuffield Department of Clinical Neurosciences, University of Oxford, Oxford, UK; 3 Division of Psychology, Communication and Human Neuroscience, School of Health Sciences Faculty of Biology, Medicine, and Health, University of Manchester, UK; 4 Wolfson Brain Imaging Centre, Department of Clinical Neurosciences, University of Cambridge, Cambridge, UK; 5 High Field MR Center, Department of Biomedical Imaging and Image-Guided Therapy, Medical University of Vienna, Vienna, Austria; 6 Aarhus University, Aarhus, Denmark; 7 Department of Physics, University of Oxford, Oxford, UK; 8 Department of Imaging Methods, Institute of Measurement Science, Slovak Academy of Sciences, Bratislava, Slovakia

**Keywords:** ^31^P spectroscopy, accelerated k-space traversal, concentric ring trajectory, myocardial energetics

## Abstract

**Purpose:**

PCr/ATP ratio is determined at 7 T typically using Fourier-transform based magnetic resonance spectroscopic imaging sequences (FT-MRSI). These sequences require acquisition times longer than desirable for inclusion in cardiac clinical trials. Concentric ring trajectory (CRT-MRSI) has been described as an accelerated alternative k-space sampling method. In this work we aim to establish the inter- and intra-session repeatability of three different CRT protocols and compare their voxel-based PCr/ATP ratios to compartment-based PCr/ATP values extracted with spectroscopy using a linear algebraic model (SLAM) method.

**Methods:**

Seven healthy volunteers were scanned twice on two different days. Each time a 6.5-min 3D FT-MRSI acquisition with 10 × 10 × 10 resolution was followed by a 2.5-min CRT-MRSI with matched resolution, a 1.5-min CRT-MRSI with matched resolution, and a 6.9-min CRT-MRSI with 12 × 12 × 12 resolution. Spectra from a mid-septal voxel and the cardiac compartment were fitted with the OXSA toolbox. PCr/ATP ratio was quantified for inter- and intra-session repeatability analysis.

**Results:**

Paired repeated measurements were not significantly different within subjects. Good inter- and intra-session agreement was observed between FT-MRSI and each CRT-MRSI protocol. CRT-MRSI protocols all had larger coefficients of repeatability (CoR) than FT-MRSI. CRT-SLAM-based PCr/ATP values had lower CoR than voxel-based data except for 2.5-min CRT-SLAM, and high-resolution CRT-SLAM had lower inter-session CoR compared to FT-MRSI (1.42 vs. 2.21).

**Conclusion:**

We established the repeatability of CRT-MRSI-based PCr/ATP values and showed higher SNR and lower CoR for CRT-SLAM. Our findings allow shorter ^31^P MRS acquisition times and the use of more advanced energetics-probing techniques in clinical studies.

## 1 Introduction

Phosphorus magnetic resonance spectroscopy (^31^P-MRS) can probe the energy metabolism of the human heart in vivo by measuring the phosphocreatine to adenosine triphosphate concentration ratio (PCr/ATP), an indicator of heart failure [1, 2]. The kinetics of the oxidative phosphorylation pathway can also be characterized using ^31^P-MRS. Short scan times are desirable irrespective of the application, with the aim of either fitting ^31^P-MRS into a clinical protocol, or to mitigate the multiple acquisitions required for chemical kinetics evaluation.

When transitioning from 3 T to 7 T, employing fast MRSI readout trajectories could allow ^31^P MRSI to leverage the full expected 2.8-fold increase in SNR [3] and hence achieve the theoretical 7.8-times (2.8^2^) speed increase when transitioning from 3 T to 7 T. This is not possible using the most common approach, the point-by-point Cartesian sampling (Fourier-transform-based MRSI, FT-MRSI). A candidate fast MRSI readout alternative, called concentric ring trajectory (CRT), has recently been described [4].

Compared to FT-MRSI, the CRT readout allows the measurement of PCr/ATP maps in a fraction of the time with matched resolution or with higher spatial resolution in matched time. While the repeatability of cardiac 3D ^31^P-MRSI sequences using Cartesian sampling at 7 T is known [5], the repeatability of the CRT sequence is yet to be determined.

Despite the attractive features of CRT-MRSI, data interpretation may still be subject to the way relevant septal cardiac voxels are selected [5]. Localized spectroscopy using a linear algebraic model (SLAM) allows the selection of anatomical compartments that contribute to the ^31^P signal [6], thus minimizing any variation in the measured PCr/ATP due to suboptimal voxel selection, effectively permitting the acquisition of spectra from arbitrarily shaped compartments [7].

Therefore, the joint aims of this study were to (1) evaluate the intra- and inter-session repeatability of three different CRT protocols, and (2) evaluate three different CRT protocols combined with compartment-based reconstruction of ^31^P spectra for a fast and robust determination of PCr/ATP values.

## 2 Methods

### 2.1 Data Acquisition

Seven healthy participants (2 females, 66 ± 9 kg, 30 ± 6 years) were scanned in supine position on a whole-body Siemens Magnetom 7 T scanner (Siemens, Erlangen, Germany). All participants were scanned under an institutionally approved technical development standard operating procedure. A 10 cm transmit/receive single-loop ^1^H coil was used to localize the heart (Rapid Biomedical, Rimpar, Germany). Without changing the participants’ position on the scanner table, the ^1^H coil was replaced with a square surface transmit coil and a 16-channel receive array coil (both by Rapid Biomedical, Rimpar, Germany) for the ^31^P acquisitions.

After localization, four sequences were run:

- Fourier transform-based acquisition-weighted MRSI (FT-MRSI) at 10 × 10 × 10 matrix size with 4 averages (at center of k-space) of 6 min and 31 s length;
- CRT-MRSI sequence with 10 × 10 × 10 matrix size employing 12 rings and 12 partitions in the z direction (2 min 31 s);
- CRT-MRSI sequence with 10 × 10 × 10 matrix size employing 10 rings and 10 partitions in the z direction (1 min 37 s);
- CRT-MRSI sequence with 12 × 12 × 12 matrix size employing 19 rings and 19 partitions in the z direction (6 min 55 s), labeled as high-res CRT-MRSI or HR-CRT-MRSI.

CRT-MRSI acquisitions were density weighted both in the *x*–*y* plane and the *z*-direction and up to two temporal interleaves were used to overcome spectral bandwidth limitations [4]. Other sequence parameters were as follows: 240 × 240 × 200 mm^3^ FoV, TR = 1 s, spectral bandwidth 8 kHz, and 2048 time-samples were used for FT-MRSI, while CRT-MRSI had matched FoV and TR, but 2778 Hz spectral bandwidth with 720 time-samples. No B_0_-shimming was performed for the FT-MRSI and CRT-MRSI acquisitions. All data was acquired without cardiac gating.

**Figure 1:**
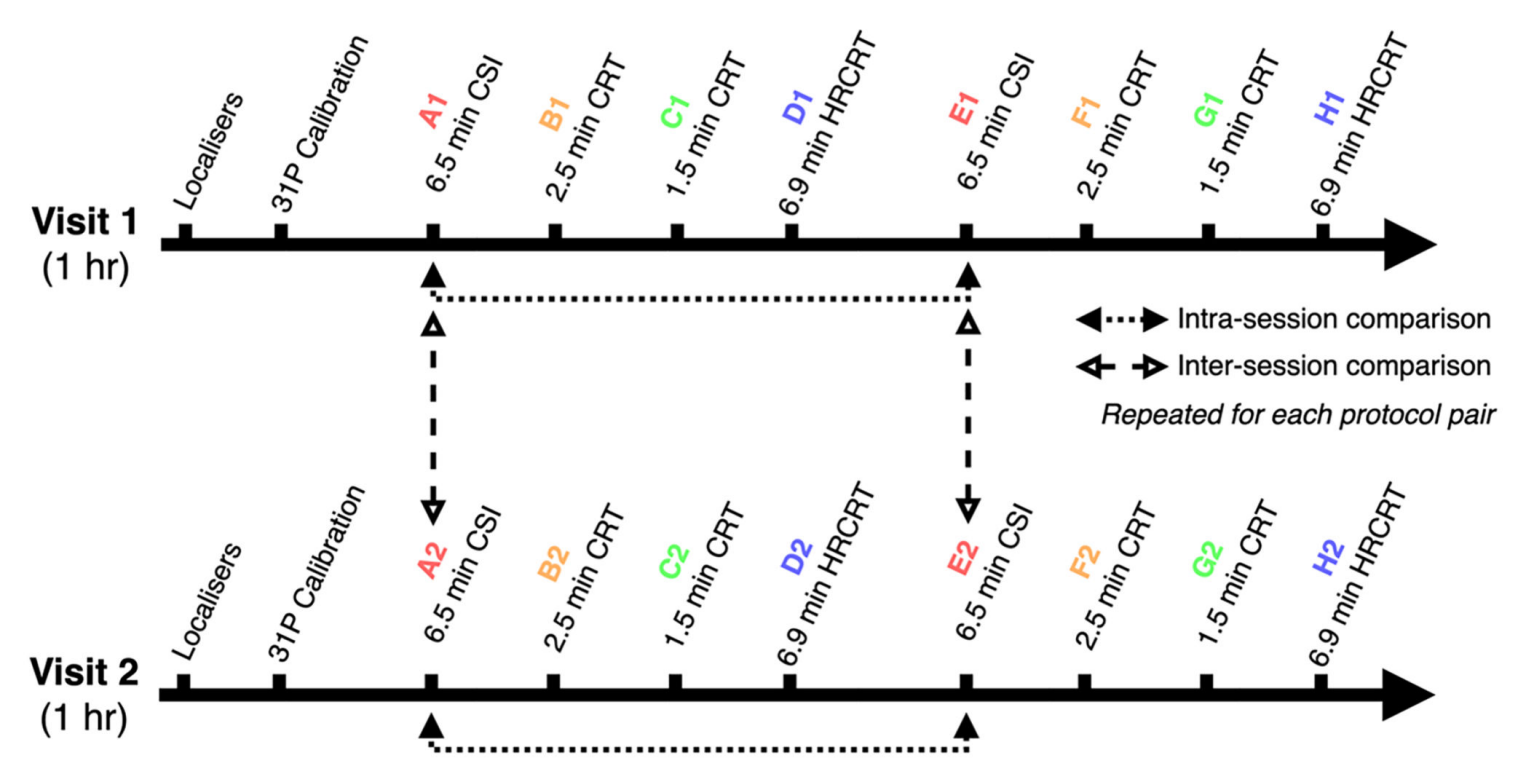
Study protocol and timings of spectroscopy acquisitions. Study participants completed two study visits during which the same four sequences (A1, B1, C1, D1) were repeated twice.

Each of the four sequences was run twice within a session, and repeated (twice) during a second study visit 72 h later, to evaluate intra- and inter-session repeatability. Figure 1 shows the schematic of the scan protocol timings, denoting the different acquisition during the first visit with A1 through H1, and those during the second visit from A2 through H2.

### 2.2 Data Reconstruction

FT-MRSI data were reconstructed online and CRT-MRSI data was reconstructed offline using the non-uniform FFT (NUFFT) toolbox with min-max Kaiser-Bessel kernel interpolation and two-fold oversampling [8] in MATLAB R2018b (MathWorks, Natick, MA, USA). No density compensation was applied. Individual coil data was combined using the WSVD algorithm [9]. Two compartment masks were defined: one over the myocardium and one over every other area. Regridded k-space data and a compartment mask of the myocardium drawn on a central localizer slice were input into the SLAM algorithm to produce a single, SNR-maximized ^31^P myocardial spectrum. Spectra were fitted using the AMARES algorithm implemented in the OXSA tool-box [10].

Sequences were compared using PCr/ATP ratios corrected for partial saturation and blood contamination using T_1_ values from the literature [3, 11], in mid-septal voxels from mid slices of the heart for non-uniform fast Fourier-transform (NUFFT)-reconstructed spectra, and in the myocardial compartment-specific spectrum reconstructed by SLAM. All corrections were calculated per-subject and per-session [3].

### 2.3 Statistical Analyses

The intra-session variability was assessed through the mean and difference between PCr/ATP ratios from equivalent datasets within the same session, for example, by comparison of datasets A1 with E1 and A2 with E2 of each volunteer for the FT-MRSI acquisitions. The inter-session variability was assessed through the mean and difference between PCr/ATP ratios from equivalent datasets in both protocols for each subject, for example, by comparing datasets A1 with A2, and E1 with E2 in case of FT-MRSI.

The coefficient of repeatability (CoR) was calculated from the average variance of within-participant PCr/ATP ratios according to Equation (1), where m_1i_ and m_2i_ represent the first and second measurement, respectively, obtained from the *i*th participant. A lower CoR reflects better repeatability.

(1) $$\text{CoR=}\sqrt{\Sigma_{i=1}^{N}\frac{{\left(m_{1i}-m_{2i}\right)}^{2}}{2N}}\times \sqrt{2}\times 1.96=\sqrt{{\mathrm{var}}_{\text{intra−subject}}}\times \sqrt{2}\times 1.96$$

Absolute reproducibility was determined using Equation (2). A lower absolute reproducibility reflects better reproducibility.

(2) $$AR[\%]=\frac{\text{COR}}{{\text{PCr/ATP}}_{\text{mean}}}\times 100$$

For the comparison of voxel-based and compartment-based PCr/ATP values, the single voxel analysis of the NUFFT-reconstructed 2.5-min CRT-MRSI acquisition was used as a reference measurement and this was compared against the 1.5-min CRT-MRSI and HR-CRT-MRSI acquisitions, as well as the three SLAM-reconstructed variants. Agreement with the NUFFT-reconstructed 2.5-min CRT-MRSI acquisition was assessed using Wilcoxon signed rank tests with Bonferroni-Holm correction for multiple comparisons, and Bland–Altman plots between SLAM-reconstructed acquisitions and the 2.5-min CRT-MRSI with NUFFT acquisition.

## 3 Results

### 3.1 Repeatability of CRT-MRSI Acquisitions

**Figure 2:**
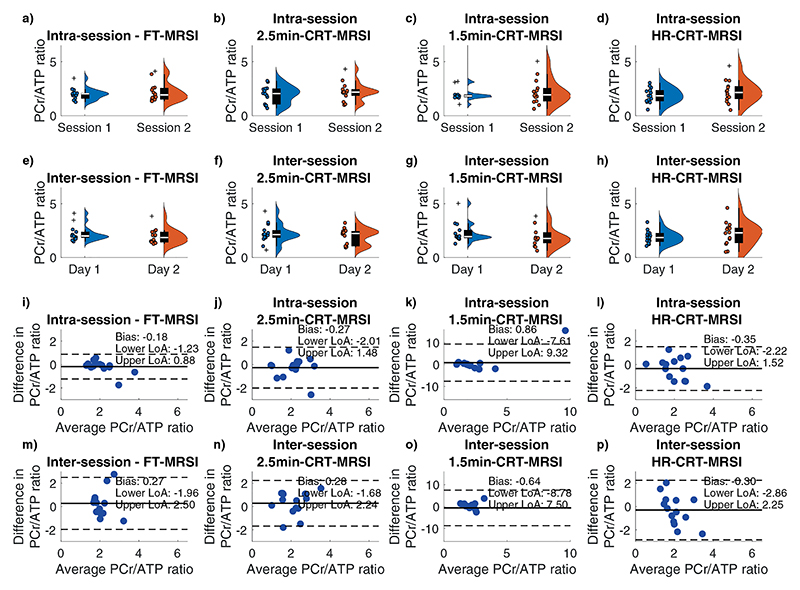
Intra-session (a–d) and inter-session (e–h) corrected PCr/ATP ratio of mid septal voxels for each sequence type: FT-MRSI (a, e), 2.5 min CRT-MRSI (b, f), 1.5 min CRT-MRSI (c, g), and HR-CRT-MRSI (d, h). Distribution estimates are shown next to individual data points. Bland– Altman analysis of intra- (i–l) and inter-session (m–p) variability in PCr/ATP for all sequence types in mid-septal voxels: FT-MRSI (i, m), 2.5 min CRT-MRSI (j, n), 1.5 min CRT-MRSI (k, o), and HR-CRT-MRSI (l, p). Solid black lines show the bias from zero; dashed lines mark lower and upper limits of agreements (bias ±1.96 × SD of the differences). Cross symbols on plots a–h represent outliers.

A typical position of the analyzed voxels as well as representative spectra from the mid-septal voxel of one participant demonstrating good agreement between the acquired data for all sequences is shown on Figure S1. Wilcoxon signed rank tests showed no significant differences in the estimated cardiac PCr/ATP between repeated measurements for all voxels, sequences, and repetitions (Figure 2a–h). The Bland–Altman plots showing the repeatability of all sequences in mid-septal voxels for inter- and intra-session measurements are depicted in Figure 2i–p. These plots show good intra-session agreement for all four acquisitions (|bias| < 0.9), as well as acceptable inter-session agreement (|bias| < 0.7).

High repeatability was observed for the long FT-MRSI scan with CoR for the mid-septal voxel of 1.07 for intra-session and 2.21 for inter-session comparison. With a shorter acquisition time, the CRT protocols sacrificed signal-to-noise (SNR) compared to the FT-MRSI, leading to higher CoR (1.83) for the intra-session but not for the inter-session comparison (CoR: 2.12) of the mid-septal voxel of the 2.5-min CRT-MRSI sequence. Table 1 presents the observed coefficients of repeatability for all four sequences.

### 3.2 Comparison of Voxel-Based and Compartment-Based PCr/ATP Estimates

**Figure 3:**
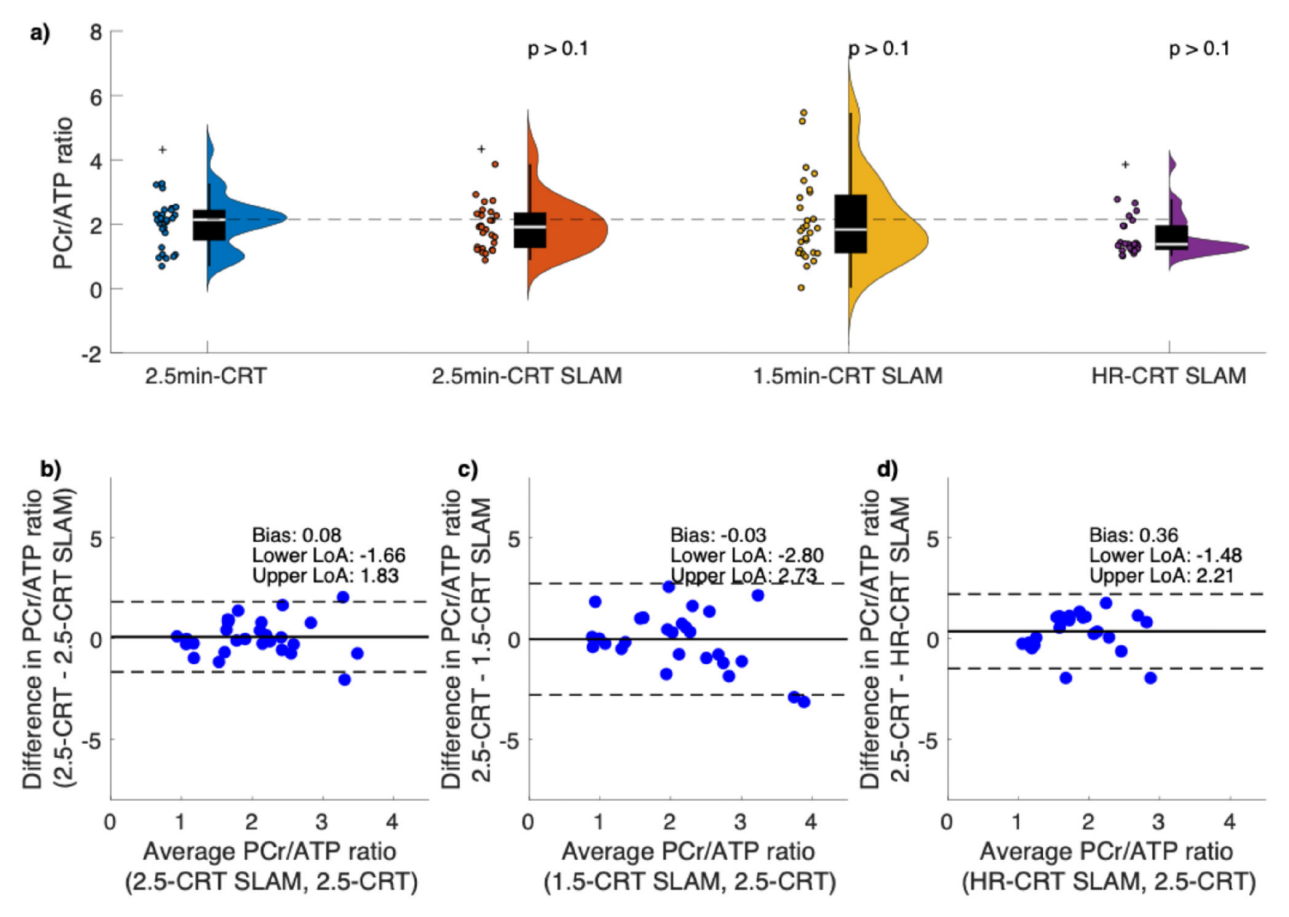
Distribution of PCr/ATP ratios from NUFFT- and SLAM-reconstructed CRT sequences (a). Bonferroni-Holm corrected *p* values above graphs correspond to Wilcoxon rank sum tests against the PCr/ATP ratios from the 2.5-min NUFFT-reconstructed CRT acquisition. The dashed line corresponds to the median PCr/ATP ratio of the 2.5-min CRT-MRSI measurements. Bland–Altman plots show the level of agreement between 2.5-min CRT-SLAM (b), 1.5-min CRT-SLAM (c), 6.9-min CRT-SLAM (d) and 2.5-min CRT. Solid black lines show the bias from zero; dashed lines mark lower and upper limits of agreement (bias ±1.96 × 1 SD of the differences). Cross symbols on plot a represent outliers.

One data set was excluded from compartment-based analysis due to missing matched localizer images needed by the SLAM algorithm. Example spectra from both NUFFT- and SLAM-reconstructed 2.5-min CRT-MRSI acquisition are shown on Figure S2. Wilcoxon signed rank tests showed no significant differences in the estimated cardiac PCr/ATP between repeated measurements for all compartments, sequences, and repetitions (Figure S3a–f). Bland–Altman plots suggest good intra- and inter-session repeatability of all compartment-based reconstructions (Figure 3g–l). Neither PCr/ATP ratio determined from SLAM-reconstructed spectra, nor the NUFFT-reconstructed spectra were significantly different from the 2.5-min NUFFT-reconstructed CRT-MRSI acquisition (Table 1 and Figure 3a). There was good agreement between the NUFFT-reconstructed 2.5-min CRT-MRSI acquisition and all the SLAM-reconstructed PCr/ATP ratios (0.08, −0.03, and 0.36 for 2.5-min CRT-SLAM, 1.5-min CRT-SLAM, and HR-CRT-SLAM, respectively) (Figure 3b–d). Similarly good agreements were observed between the NUFFT-reconstructed 1.5-min CRT-MRSI acquisition and all the SLAM-reconstructed PCr/ATP ratios (Figure S4), as well as between the NUFFT-reconstructed HR-CRT-MRSI acquisition and all the SLAM-reconstructed PCr/ATP ratios (Figure S5). Most SLAM-reconstructed PCr/ATP ratios had lower (better) coefficients of repeatability than corresponding MRSI-reconstructed PCr/ATP ratios, with the exception of FT-SLAM, and the intra-session repeatability of 2.5-min-CRT-SLAM.

## 4 Discussion

In this work we established the repeatability of cardiac PCr/ATP ratios measured with a 3D CRT sequence employing MRSI and SLAM reconstruction at 7 T in a group of young healthy volunteers. We found that PCr/ATP from all CRT-MRSI protocols had generally higher coefficients of repeatability than the PCr/ATP from the standard FT-MRSI sequence, owing to the trade-off of SNR in favor of gains in time acceleration or increased spatial resolution by the CRT-MRSI sequences. When comparing CRT-SLAM sequences against the 2.5-min CRT-MRSI sequence, we found an increase in the SNR of PCr.

Our findings are in line with previous reports. The FT-MRSI absolute reproducibility (51%) is comparable with the absolute reproducibility of PCr/ATP measured using 3D FT-MRSI as determined both at 3 T and 7 T, despite the shorter acquisition time. Tyler et al. [11] reported 53% absolute reproducibility at 3 T, while Ellis et al. [5] found 39% absolute reproducibility at 7 T using a comparable 3D FT-MRSI protocol. While the 2.5-min CRT-MRSI and HR-CRT-MRSI sequences had near-identical absolute reproducibility, the extreme shortening of the acquisition time to 1.5 min resulted to be unfeasible using voxel-based reconstruction.

Whilst having an accelerated 3D ^31^P acquisition available is already a great step towards more patient-friendly clinical cardiac MRI protocols, performing a SLAM reconstruction instead of the standard MRSI reconstruction can demonstrably increase SNR and increase repeatability. Inter-reader variability of PCr/ATP measurements is introduced by manual selection of cardiac voxels from 3D acquisitions. This variability might be further reduced by performing an automatic segmentation of the myocardium, thus allowing for the automatic extraction of PCr/ATP ratios.

We note the consistently lower PCr/ATP ratio by HR-CRT-SLAM compared to other sequences and reconstruction methods. It is likely that the improvement of the PSF from the smaller voxels and the SLAM reconstruction, both of which were shown to contribute to the decrease of contamination from high (skeletal muscle) and low (blood or liver) PCr/ATP compartments, produced this difference in PCr/ATP ratios.

The good repeatability metrics of the CRT sequence can be leveraged not only for direct measurements of cardiac energetics, but we expect also importantly for precise mapping of the B_1_^+^ field necessary for the assessment of the rates of chemical reactions involved in energy metabolism, namely determining ATP production rate constants via ^31^P-MRS using the Bloch-Siegert four Angle Saturation Transfer [12] method. Furthermore, compartment-based absolute quantitation of PCr and ATP concentrations [13] could also benefit from accelerated k-space traversal via concentric rings.

When compared against single-voxel spectroscopy, compartment-based reconstruction has a clear clinical advantage, as it allows the contouring of the heart, removing the variability introduced by voxel positioning in the interventricular septum [14]. It also allows the post hoc re-processing using updated contours. In addition, MRSI-type acquisitions are already preferred over single-voxel spectroscopy in many research settings. In terms of differences in scan duration, ISIS-type acquisitions would need similar acquisition times as our 2.5-min CRT sequence, given the need for long TRs and for more averages to increase SNR to a level comparable to a CRT acquisition’s SLAM reconstruction. Compartment-based reconstruction benefits from anatomically-defined resolution that is more specific than a single, potentially small voxel. Artifacts are also more prevalent in single-voxel spectroscopic acquisitions, which in turn impact spatial definition and localization of the selected voxel as well as the quality of the resulting spectra (e.g., T_1_ smearing in ISIS).

A limitation of this study is the inherent B_1_^+^ variability observed at 7 T, dependent on coil loading [15]. The flip angles achieved in the myocardium were likely not homogeneous in our healthy volunteers, leading to even higher variation in SNR and PCr/ATP ratios in obese patient populations. Reproducibility might therefore need to be re-assessed in specific disease groups. Alternatively, a whole-body ^31^P transmit coil may be used to ensure the homogeneity of the B_1_^+^ field [16]. The use of such a coil would also contribute to higher SNR and ultimately lower coefficients of repeatability.

In addition, we noticed line broadening in SLAM-reconstructed spectra, which can be ascribed to the increased size of the volume of interest from which the ^31^P signal was “averaged”, to the inclusion of parts of the myocardium that are more mobile than the septum, as well as the B_0_ field inhomogeneity, which could be minimized with B_0_ shimming.

In conclusion, we showed favorable repeatability of the PCr/ATP measured using CRT MRSI and SLAM readouts in healthy volunteers. The combination of SLAM reconstruction and CRT acquisition allows the recovery of FT-MRSI levels of reproducibility while retaining the high speed. Our findings contribute to the development of accelerated ^31^P-MRS protocols for the increased tolerability of cardiac clinical studies at 7 T, with the possible outlook for higher spatial resolution scans allowing for regional characterization of the myocardium.

## Supplementary Material

Suppl

## Figures and Tables

**Table 1:** Summary statistics of the mid-septal PCr resonance and coefficients of reproducibility of mid-septal PCr/ATP for each of the four examined acquisitions both with MRSI and SLAM reconstruction. Values are shown as mean ± standard deviation.

Technique	PCr/ATP	PCr SNR	PCr linewidth [Hz]	PCr CRLB [%]	CoR intra	CoR inter	CoV intra	CoV inter	AR intra [%]	AR inter [%]
6.5 min FT-MRSI	2.09 ± 0.71	19.6 ± 13.5	29.2 ± 10.6	15.5 ± 9.2	1.07	2.21	0.07	0.19	51.4	105.7
6.5 min FT-SLAM	2.37 ± 1.22	21.5 ± 14.1	43.3 ± 19.9	10.7 ± 33.8	1.19	3.61	0.11	0.23	50.0	152.1
2.5 min CRT-MRSI	2.07 ± 0.82	15.8 ± 9.4	35.1 ± 13.1	12.2 ± 8.5	1.83	2.12	0.14	0.21	88.7	102.4
2.5 min CRT-SLAM	1.99 ± 0.80	28.5 ± 17.4	57.4 ± 22.1	13.6 ± 22.7	2.00	1.83	0.16	0.32	100.5	92.0
1.5 min CRT-MRSI	1.93 ± 0.58	11.1 ± 7.2	33.4 ± 13.4	23.1 ± 20.0	8.33	7.94	0.22	0.29	316.9	302.2
1.5 min CRT-SLAM	2.10 ± 1.27	21.7 ± 13.4	56.6 ± 22.6	17.6 ± 31.1	1.92	2.73	0.24	0.44	91.3	129.7
HR-CRT-MRSI	2.09 ± 1.07	15.7 ± 14.6	40.0 ± 31.0	10.0 ± 7.5	1.93	2.53	0.18	0.27	94.0	123.7
HR-CRT-SLAM	1.64 ± 0.67	21.7 ± 9.3	57.0 ± 30.5	9.9 ± 4.3	1.37	1.42	0.11	0.16	83.7	91.3

Abbreviations: AR—absolute reproducibility; ATP—adenosine triphosphate; CoR—coefficient of repeatability; CoV—coefficient of variation; CRLB—Cramer-Rao lower bound; CRT—concentric ring trajectory; HR—high-resolution; MRSI—magnetic resonance spectroscopic imaging; PCr—phosphocreatine; SLAM—spectroscopy with linear algebraic modeling; SNR—signal-to-noise ratio.

## Data Availability

The data that support the findings of this study are available from the corresponding author upon reasonable request.
