# Supplementary material for: Repeatability of Rapid Human Cardiac Phosphorus MRSI ( 31P‐MRSI) Using Concentric Ring Trajectory Readouts at 7 T
Source: Magn Reson Med. 2025 Dec 8;95(5):2508–14. doi: 10.1002/mrm.70220 (PMC7618768; doi:10.1002/mrm.70220)
Supplement: Supplementary file 1 — Figure S1: Representative CSI grid positioning (A, B, and C) and spectra (D) from all four sequences measured in a mid‐septal voxel on a mid‐slice of a single participant. Each panel shows the repeated measurements within a single session. The real part of 31P spectra were normalized to have equal PCr amplitudes. Figure S2: Localization of the myocardial signal using SLAM (a) is achieved by considering all voxels within the myocardial tissue compartment and their contribution to the 31P signal, as opposed to the mid‐septal voxel‐based localization (c) prone to operator variability. The resulting real 31P spectrum from SLAM (b) has higher SNR than the real 31P spectrum from the regridded, NUFFT‐reconstructed CRT acquisition. Spectra were normalized to have equal PCr amplitudes. Yellow rectangles represent saturation bands placed over the chest muscle. Figure S3: Intra‐session (a‐d) and inter‐session (e‐h) corrected PCr/ATP ratios from the SLAM reconstruction of each sequence type: FT (a, e), 2.5 min CRT (b, f), 1.5 min CRT (c, g), and HR‐CRT (d, h). Distribution estimates are shown next to individual data points. Bland–Altman analysis of intra‐ (i–l) and inter‐session (m–p) variability in PCr/ATP for all sequence types in mid‐septal voxels: FT (i, m), 2.5 min CRT (j, n), 1.5 min CRT (k, o), and HR‐CRT (l, p). Solid black lines show the bias from zero (the mean of the signed differences in PCr/ATP ratios); dashed lines mark lower and upper limits of agreements (bias ±1.96 × SD of the differences). Cross symbols on plots a–h represent outliers. Figure S4: Distribution of PCr/ATP ratios from NUFFT‐ and SLAM‐reconstructed CRT sequences (a). Bonferroni‐Holm corrected p values above graphs correspond to Wilcoxon rank sum tests against the PCr/ATP ratios from the 1.5‐min NUFFT‐reconstructed CRT acquisition. The dashed line corresponds to the median PCr/ATP ratio of the 1.5‐min CRT‐MRSI measurements. Bland–Altman plots show the level of agreement between 2.5‐min CRT‐SLAM (b) [file MRM-95-2508-s001.docx]

Supporting information for Repeatability of rapid human cardiac phosphorus MRS (^31^P-MRS) using concentric ring trajectory readouts at 7 T

Ferenc E. Mózes, William T. Clarke, Andrew Tyler, Jabrane Karkouri, Fabian Niess, Jack J. J. J. Miller, Christopher T. Rodgers, Wolfgang Bogner, Ladislav Valkovič


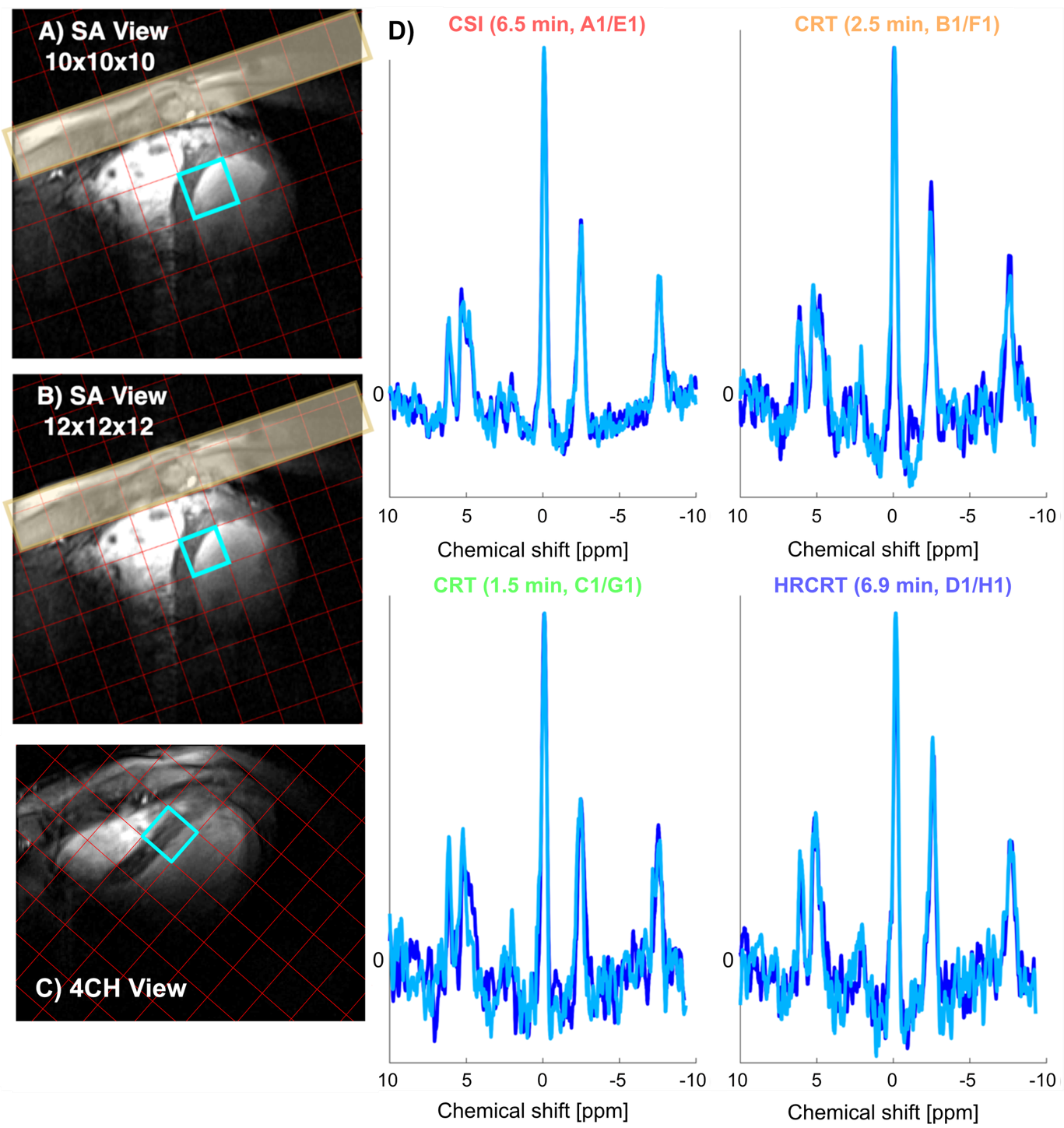


**Supporting Figure 1** Representative CSI grid positioning (A, B, and C) and spectra (D) from all four sequences measured in a mid-septal voxel on a mid-slice of a single participant. Each panel shows the repeated measurements within a single session. The real part of ^31^P spectra were normalised to have equal PCr amplitudes.


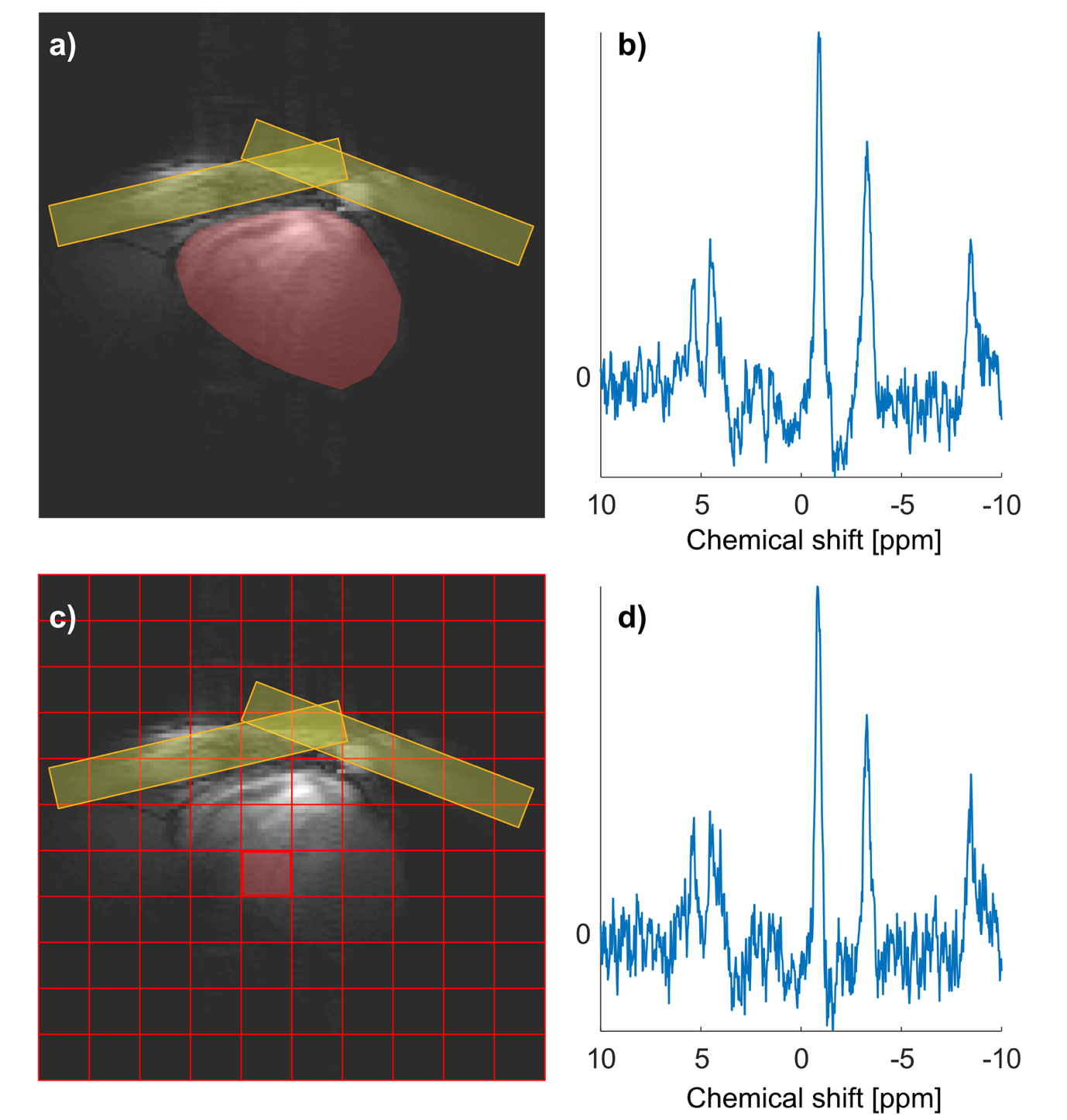


**Supporting Figure 2** Localisation of the myocardial signal using SLAM (a) is achieved by considering all voxels within the myocardial tissue compartment and their contribution to the ^31^P signal, as opposed to the mid-septal voxel-based localisation (c) prone to operator variability. The resulting real ^31^P spectrum from SLAM (b) has higher SNR than the real ^31^P spectrum from the regridded, NUFFT-reconstructed CRT acquisition. Spectra were normalised to have equal PCr amplitudes. Yellow rectangles represent saturation bands placed over the chest muscle.


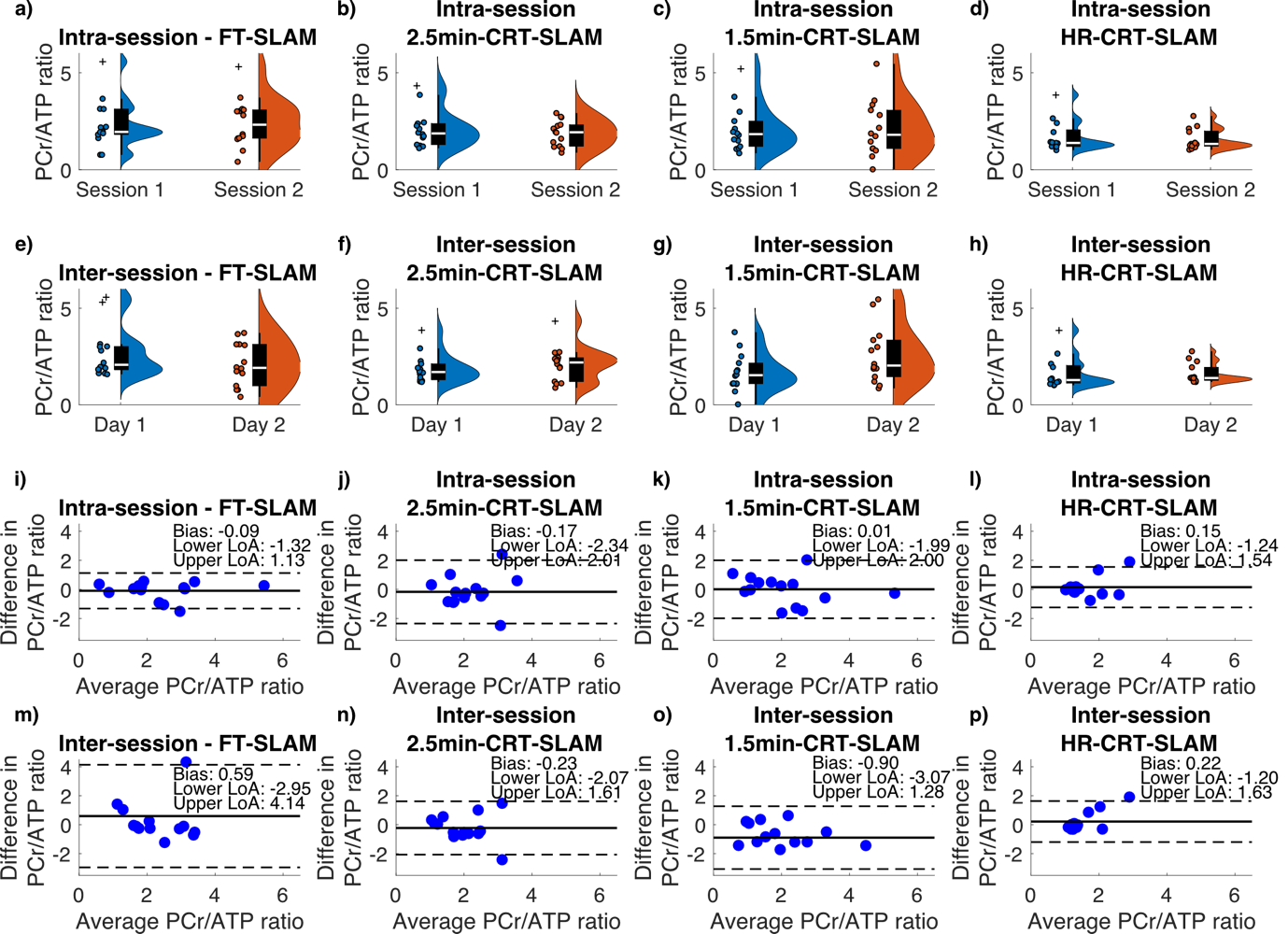


**Supporting Figure 3** Intra-session (a-d) and inter-session (e-h) corrected PCr/ATP ratios from the SLAM reconstruction of each sequence type: FT (a, e), 2.5 min CRT (b, f), 1.5 min CRT (c, g), and HR-CRT (d, h). Distribution estimates are shown next to individual data points. Bland-Altman analysis of intra- (i-l) and inter-session (m-p) variability in PCr/ATP for all sequence types in mid-septal voxels: FT (i, m), 2.5min CRT (j, n), 1.5min CRT (k, o), and HR-CRT (l, p). Solid black lines show the bias from zero (the mean of the signed differences in PCr/ATP ratios); dashed lines mark lower and upper limits of agreements (bias ± 1.96 × SD of the differences). Cross symbols on plots a-h represent outliers.

**Supporting Figure 4** Distribution of PCr/ATP ratios from NUFFT- and SLAM-reconstructed CRT sequences (a). Bonferroni-Holm corrected p-values above graphs correspond to Wilcoxon rank sum tests against the PCr/ATP ratios from the 1.5-minute NUFFT-reconstructed CRT acquisition. The dashed line corresponds to the median PCr/ATP ratio of the 1.5-minute CRT-MRSI measurements. Bland-Altman plots show the level of agreement between 2.5-minute CRT-SLAM (b), 1.5-minute CRT-SLAM (c), 6.9-minute CRT-SLAM (d) and 1.5-minute CRT. Limits of agreement are wider than the figures may suggest otherwise due to a single outlier on each of the plots (outlier not shown; average PCr/ATP of 9.86, 9.73, and 9.36, and difference in PCr/ATP of 14.96, 15.23, and 15.96, corresponding to panels b, c, and d, respectively). Solid black lines show the bias from zero (the mean of the signed differences in PCr/ATP ratios); dashed lines mark lower and upper limits of agreement (bias ± 1.96 × 1 SD of the differences). Cross symbols on plot a represent outliers.

**Supporting Figure 5** Distribution of PCr/ATP ratios from NUFFT- and SLAM-reconstructed CRT sequences (a). Bonferroni-Holm corrected p-values above graphs correspond to Wilcoxon rank sum tests against the PCr/ATP ratios from the high-resolution NUFFT-reconstructed CRT acquisition. The dashed line corresponds to the median PCr/ATP ratio of the HR-CRT-MRSI measurements. Bland-Altman plots show the level of agreement between 2.5-minute CRT-SLAM (b), 1.5-minute CRT-SLAM (c), 6.9-minute CRT-SLAM (d) and HR-CRT. Solid black lines show the bias from zero (the mean of the signed differences in PCr/ATP ratios); dashed lines mark lower and upper limits of agreement (bias ± 1.96 × 1 SD of the differences). Cross symbols on plot a represent outliers.
